# Paroxysmal Hæmoglobinuria

**Published:** 1887-02

**Authors:** F. R. Campbell


### *PAROXYSMAL HÆMOGLOBINURIA.*

Translated from *Nordiskt Medicinskt Arkiv.*, by F. R. CAMPBELL, M. D.

R. Bruzelius, of Stockholm, gives the clinical history of three cases of this disease which he has observed in the Swedish hospitals These are the only cases that have been observed in Sweden, and only two have been reported in Norway. Lichtheim, in an article on paroxysmal hæmoglobinuria, mentions two cases which have been observed in Germany; and since the publication of his monograph several other cases have been recorded. The disease is more common in England and France. Murri, of Italy, has also reported several cases. Both sexes are affected and it is met with at all ages, from the infant of two years to the old man of seventy. The first case reported by Bruzelius was that of a woman, aged 27 years, who was attacked with the disease after prolonged exposure to cold. Slight chills, pain in the sacral region, prickling of the skin and urticaria accompanied the disease or preceded it. On two subsequent occasions the malady returned each time after exposure to cold.. In one attack the skin became icteric, and it was always observed that the intensity of the attacks was in direct ratio with the severity of the exposure to cold. The sensibility of the patient to cold was quite remarkable. If the hand was held in a cold draft, while the body was protected in a warm room, urticaria was promptly developed on the exposed skin.

The crisis of the disease was always accompanied by chills, elevation of temperature (103° in rectum), perspiration, pain in sacral region, urticaria, icterus, frequent micturition and a heavily-clouded urine, containing albumen, casts and hæmoglobin, but no blood corpuscles. The fever disappears in from three to eight hours, when the urine assumes its normal appearance. In some of the milder attacks, there was a slighter elevation of temperature, and although the urine contained albumen no hæmoglobin was detected. A microscopic examination of the blood in hæmoglinuria reveals no traces of destruction of blood corpuscles, but the spectroscope demonstrates the presence of hæmoglobin. In March, 1886, twelve years after the first attack, Bruzelius saw this patient. She had had no attack in three years, as she was very careful to avoid exposure to cold, but whenever, by any chance, she became slightly chilled the urticaria returned. In other respects, she was in good health.

The second case was that of a man fifty-two years of age, who had had many times during the preceding five years attacks of hæmoglobinuria following exposure to cold. A host of remedies were tried without success, and the disease continued during the remaining eight years of the patient's life, when he died of fatty degeneration of the heart. There were no evidences of syphilis in the case.

The third case was that of a man, seventy-two years of age, whose disease continued two years when he died of acute pneumonia.

The writer believes the disease to be an affection of the blood where the destruction of the corpuscles is going on in the organs of circulation, and not simply a disease of the kidneys, as is taught by Rosenbach. Bruzelius' cases all resulted from exposure to cold, but other observers have seen it follow bodily fatigue, such as marching. Murri and Schumacher consider syphilis the true cause of the disease. One of the cases of the writer was syphilitic, but there was no evidence of such a complication in either of the other patients.

